# Evaluation of Severity Levels of the Athens Insomnia Scale Based on the Criterion of Insomnia Severity Index

**DOI:** 10.3390/ijerph17238789

**Published:** 2020-11-26

**Authors:** Isa Okajima, Towa Miyamoto, Ayaka Ubara, Chie Omichi, Arichika Matsuda, Yukiyoshi Sumi, Masahiro Matsuo, Kazuki Ito, Hiroshi Kadotani

**Affiliations:** 1 Department of Psychological Counseling, Faculty of Humanities, Tokyo Kasei University, Tokyo 173-8602, Japan; 2 Department of Sleep and Behavioral Sciences, Shiga University of Medical Science, Shiga 520-2192, Japan; cykc1005@mail2.doshisha.ac.jp (A.U.); comichi@koto.kpu-m.ac.jp (C.O.); arichika@belle.shiga-med.ac.jp (A.M.); momo3@belle.shiga-med.ac.jp (K.I.); kadotanisleep@gmail.com (H.K.); 3 Department of Psychiatry, Shiga University of Medical Science, Shiga 520-2192, Japan; twmymt@belle.shiga-med.ac.jp (T.M.); elasticvisco@gmail.com (Y.S.); mazzuo@belle.shiga-med.ac.jp (M.M.); 4 Graduate School of Psychology, Doshisha University, Kyoto 610-0394, Japan; 5 JSPS Research Fellowships, Tokyo 102-0083, Japan; 6 Department of Psychiatry, Graduate School of Medical Science, Kyoto Prefectural, University of Medicine, Kyoto 602-8566, Japan; 7 Department of Anesthesiology, Shiga University of Medical Science, Shiga 520-2192, Japan

**Keywords:** Athens Insomnia Scale, Insomnia Severity Index, depression, quality of life, cutoff, sleepiness

## Abstract

The Athens Insomnia Scale (AIS) can be regarded as a highly useful instrument in both clinical and research settings, except for when assessing the severity level. This study aims to determine the severity criteria for AIS by using the Insomnia Severity Index (ISI). A total of 1666 government employees aged 20 years or older were evaluated using the AIS and ISI, the Patient Health Questionnaire for depressive symptoms, the Epworth Sleepiness Scale for daytime sleepiness, and the Short Form Health Survey of the Medical Outcomes Study for health-related quality of life (QoL). A significant positive correlation (*r*) was found between the AIS and the ISI (*r* = 0.80, *p* < 0.001). As a result of describing receiver–operator curves, the severity criteria of the AIS are capable of categorizing insomnia severity as follows: absence of insomnia (0–5), mild insomnia (6–9), moderate insomnia (10–15), and severe insomnia (16–24). In addition, compared to all scales across groups categorized by AIS or ISI, it was revealed that similar results could be obtained (all *p* < 0.05). Therefore, the identification of the severity of AIS in this study is important in linking the findings of epidemiological studies with those of clinical studies.

## 1. Introduction

Nearly 20% of the general adult population in Japan has been reported to demonstrate symptoms related to insomnia [1,2], and it is known that 5–19% of individuals have insomnia that follows a chronic course [3,4]. In addition, insomnia severity has a great association with depression severity and symptoms [5], and sleep disturbances increase the risk of sick leave up to 23% [6]. Currently, cognitive behavioral therapy (CBT-I) is recommended as a treatment for chronic insomnia [4,7].

There are several self-rating scales for insomnia severity currently available for evaluating subjective insomnia. Among these, the Insomnia Severity Index (ISI) [8,9,10] and Athens Insomnia Scale (AIS) [11,12,13] are commonly used as authorized insomnia symptom questionnaires. These scales have been shown to have appropriate diagnostic utility, and they include a set of items for evaluating both nocturnal sleep disturbance and daytime dysfunction. In particular, the ISI includes some psychological components of insomnia (e.g., worry and satisfaction), while the AIS includes physiological components (e.g., sleepiness and functioning capacity during the day). Chiu et al. [14] conducted a meta-analysis to evaluate the diagnostic accuracy of three screening tools for insomnia: the ISI, AIS, and Pittsburgh Sleep Quality Index (PSQI) [15]. They showed that these scales are useful instruments for insomnia screening, and the diagnostic accuracy of each scale (sensitivity and specificity) was high (ISI: 88% and 85%, AIS: 91% and 87%, and PSQI: 94% and 76%, respectively). In particular, they suggested that the ISI and AIS are probably stronger and appropriate instruments than the PSQI, according to the comparisons of scale characteristics for diagnostic properties, sleep domains, and feasibility [14].

With regard to the specific characteristics of each scale, the ISI is only capable of categorizing insomnia severity as follows: absence of insomnia (0 to 7 points), subthreshold (mild) insomnia (8 to 14 points), moderate insomnia (15 to 21 points), and severe insomnia (22 points or more) [8]. Therefore, the scale has been frequently utilized in clinical researches [4,7].

On the other hand, the AIS estimates nocturnal sleep disturbance and daytime dysfunctions that occur at least three times a week, on the basis of the International Classification of Diseases, 10th edition (ICD-10) criteria [11,12]. It was reported that the cutoff score is 5.5 points in both European and Asian countries [11,13], which is a common score across cultures. However, the AIS was rarely used in clinical studies [16] but frequently utilized in cohort or cross-sectional studies [17,18]. Furthermore, there is yet to be a study examining the accuracy of insomnia severity in insomnia scales, although the diagnostic accuracy of insomnia scales has been reported [14].

Thus, both AIS and ISI are considered the “gold standard” of insomnia scales and have high diagnostic accuracy. The AIS has some physical components while the ISI contains some psychological components. In addition, the AIS can be regarded as a highly useful instrument in both clinical and research settings, except for not having the ability to assess severity level. Since the AIS created the basis of the ICD-10 diagnostic criteria, more diverse findings can be found than ever before, not only in cross-sectional and cohort studies, but also in clinical trials, if the AIS is capable of assessing the severity of insomnia. Therefore, this study aimed to determine the severity criteria for AIS using ISI, which categorizes mild, moderate, and severe levels of insomnia.

## 2. Materials and Methods

### 2.1. Participations

Participants were government employees of Koka city, which is a rural city in the Shiga Prefecture of Japan [19]. Employees for whom the consent of a legal representative was required for participation or who had taken extended leave from employment were excluded. Among the 2119 prefectural government’s employments, 62 were excluded because of extended leave, including sick, maternity, and childcare leave. Thus, a total of 2057 participants were included. Responses to the questionnaire were obtained from 1685 people anonymously (response rate: 81.9%). Of them, 1666 people completely answered both AIS and ISI (649 males, 1017 females, mean age (SD): 45.33 (12.20) years old). The questionnaire survey was conducted from 6 September to 15 November 2017.

### 2.2. Measures

#### 2.2.1. Insomnia Symptoms

Insomnia symptoms were measured using the Japanese version of AIS [13], a validated eight-item self-report questionnaire that assesses insomnia symptom over the past month. A sum score is calculated (range: 0–24), with lower scores indicating fewer insomnia symptoms. A cutoff score discriminating healthy patients from those suffering from insomnia is 5.5 points [12,13]. Insomnia severity was measured using the Japanese version of ISI [10], a validated seven-item self-report questionnaire that assesses insomnia severity over the past 2 weeks. A sum score is calculated (range: 0–28), with lower scores indicating fewer insomnia symptoms. Severity level is categorized as no insomnia (0 to 7 points), subthreshold (mild) insomnia (8 to 14 points), moderate insomnia (15 to 21 points), and severe insomnia (22 points or more) [8].

#### 2.2.2. Daytime Sleepiness, Depressive Symptoms, and Quality of Life (QoL)

We measured depressive symptoms, daytime sleepiness, and QoL as insomnia-related daytime dysfunction. Depressive symptoms were measured using the Japanese version of Patient Health Questionnaire (PHQ-9) [20], a validated nine-item questionnaire that assesses depressive symptom severity over the past week. A sum score is calculated (range: 0–27), with lower scores indicating less depression. Daytime sleepiness was measured using the Japanese version of Epworth Sleepiness Scale (ESS) [21], a validated eight-item questionnaire that assesses excessive daytime sleepiness. A sum score is calculated (rage: 0–24), with lower scores indicating less sleepiness. QoL was measured using the Japanese version of Short Form Health Survey of the Medical Outcomes Study (SF-8) [22], a validated eight-item questionnaire that assesses health-related quality of life over the past week. The mental component summary (MCS) scale of the SF-8 was used to evaluate mental QoL, and the physical component summary (PCS) scale was used to evaluate physical QoL. The average scores for both scales for the general population were set to 50 points.

### 2.3. Procedures

A cross-sectional questionnaire-based study was conducted as part of the Night in Japan Home Sleep Monitoring (NinJaSleep) Study, which is an epidemiological study on sleep and mental health [19]. The study was approved by the ethics committee of the Shiga University of Medical Science (27-056). Informed consent was obtained from each participant prior to participation. We distributed a questionnaire at their workplace, and the individuals who responded to the questionnaire remained anonymous.

### 2.4. Statistics Analysis

Descriptive statistics were computed using R statistical software version 3.6.3 (R Project for Statistical Computing, Vienna, Austria). To associate between the AIS and ISI, we conducted a correlation analysis and an exploratory factor analysis utilizing a maximum-likelihood solution method with promax rotation. In the factor analysis, all items on the AIS and ISI were analyzed simultaneously. Factors were determined by setting eigenvalues to ≥1 according to the shape of the scree plot.

In addition, we described a receiver–operator curve (ROC) in order to determine severity levels of mild, moderate, and severe insomnia. The ROC was plotted, and the mean (95% confidence interval (CI)) area under the curve (AUC) was used to estimate an AIS cutoff score for distinguishing pathological insomnia from a normal condition. When the tangent line slope of the ROC is statistically equal to 1 (i.e., AUC = 0.5), then the ROC is considered inaccurate for prediction purposes. The predictive ability of a variable was classified with reference to the AUC (outstanding >0.9, excellent = 0.8–0.9, good = 0.7–0.8, acceptable = 0.6–0.7, poor = 0.5–0.6, or nondiscriminative = 0.5) [23]. The best cutoff value for pathological insomnia was determined on the basis of sensitivity, specificity, positive likelihood ratio (LR+), and negative likelihood ratio (LR−). In accordance with the authorized method, the cutoff score was assessed as adequate when LR+ was 2.0 or higher and LR− was 0.5 or less [2,24].

It was already revealed that a cutoff score discriminating normal sleep from insomnia is 5.5 points on the AIS [13]. Therefore, we described the ROC of mild vs. moderate level and that of moderate vs. severe level.

On the basis of severity level of the AIS or ISI, we categorized four groups as no, mild, moderate, and severe insomnia groups. An ANOVA was used to compare groups on each scale. When main effects in all analyses were shown, we performed Bonferroni–Holm correction for *p*-values and then conducted post hoc analyses.

## 3. Results

### 3.1. Exploratory Factor Analysis of AIS and ISI

Table 1 summarizes the participant characteristics. The correlation analysis showed a significant positive correlation (*r*) between the AIS and the ISI (*r* (95%CI) = 0.80 (0.78–0.82), *p* < 0.001). In addition, exploratory factor analysis on all items of the AIS and ISI showed a two-factor structure, namely, “nocturnal sleep” and “sleep quality and daytime function” (Table 2). The factor of “nocturnal sleep” consisted of items for initial, middle, and terminal insomnia, and the factor of “sleep quality and daytime function” included items such as sleep quality (AIS item 5), wellbeing (AIS item 6), satisfied/dissatisfied with sleep pattern (ISI item 4), and sleep problem interference with daily functioning (ISI item 5).

### 3.2. ROC to Evaluate Severity Level of the AIS

**Figure 1:**
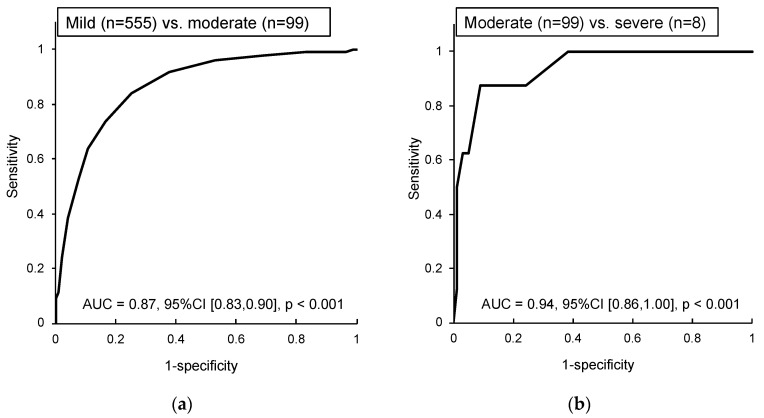
Cutoff point of the AIS for insomnia severity estimated using the receiver–operator curve (ROC): (**a**) ROC at mild vs. moderate levels; (**b**) ROC at moderate vs. severe levels.

We used the ROC to examine the cutoff value of mild, moderate, and severe levels for the AIS scores. As a result, the AUC of the ROC was 0.87 (95% CI, 0.83 to 0.90, *p* < 0.001) at a mild/moderate level and 0.94 (95% CI, 0.86 to 1.00, *p* < 0.001) at a moderate/severe level (Figure 1). Both values were statistically larger than 0.50.

The cutoff value of the AIS at mild/moderate level was estimated at 9.5 points. This cutoff value’s sensitivity was 74%, specificity was 83%, positive likelihood ratio was 4.45, and negative likelihood ratio was 0.32 (Table 3). In addition, the cutoff value of the scale at moderate/severe level was estimated at 15.5 points. This cutoff value’s sensitivity was 88%, specificity was 91%, positive likelihood ratio was 9.63, and negative likelihood ratio was 0.14 (Table 3).

Therefore, the severity criteria of the AIS are capable of categorizing insomnia severity as follows: absence of insomnia (0–5), mild insomnia (6–9), moderate insomnia (10–15), and severe insomnia (16–24).

### 3.3. Comparison of the Scales Based on the Cutoff for AIS or ISI

**Figure 2:**
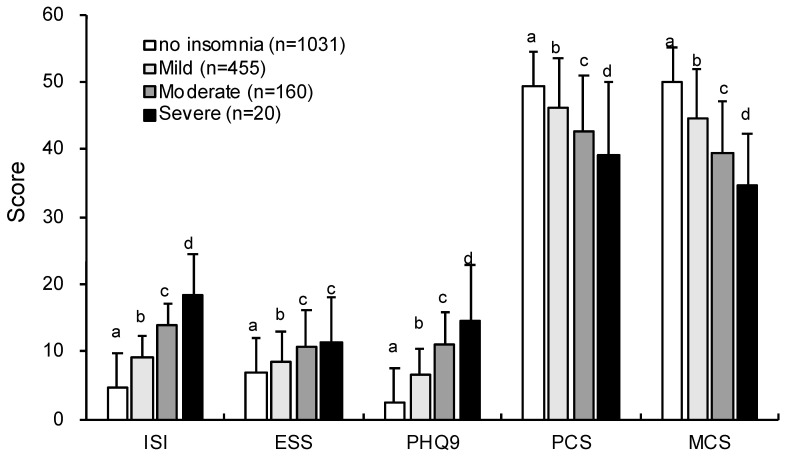
Comparison of categories for insomnia severity according to the AIS on each scale. Different letters indicate significantly different scores between groups (*p* < 0.05).

**Figure 3:**
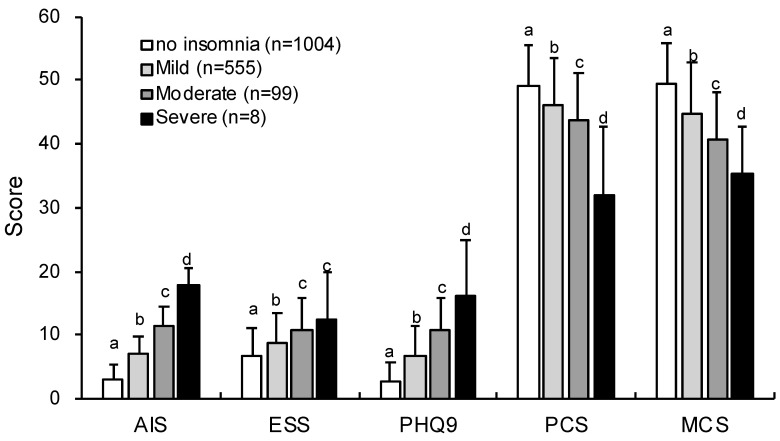
Comparison of categories for insomnia severity according to the ISI on each scale. Different letters indicate significantly different scores between groups (*p* < 0.05).

We categorized insomnia into four groups, absence of insomnia, mild insomnia, moderate insomnia, and severe insomnia, on the basis of the cutoff values for the AIS or ISI. It was revealed that similar results could be obtained using either criteria (Figure 2 and Figure 3). The scores for insomnia symptoms (i.e., ISI or AIS) and PHQ-9 were significantly higher in the order of severe, moderate, mild, and absence of insomnia (ISI cutoff: AIS (*F*_3,1665_ = 581.84, *p* < 0.001), PHQ-9 (*F*_3,1664_ = 275.40, *p* < 0.001); AIS cutoff: ISI (*F*_3,1665_ = 648.62, *p* < 0.001); PHQ-9 (*F*_3,1664_ = 423.28, *p* < 0.001)). The scores of ESS were higher for moderate and severe insomnia than for mild insomnia and absence of insomnia (ISI cutoff: *F*_3,1665_ = 43.86, *p* < 0.001; AIS cutoff: *F*_3,1665_ = 49.08, *p* < 0.001). The scores of PCS and MCS of SF-8 were lower in the order of severe, moderate, mild, and absence of insomnia (ISI cutoff: PCS (*F*_3,1654_ = 52.03, *p* < 0.001); MCS (*F*_3,1654_ = 93.66, *p* < 0.001); AIS cutoff: PCS (*F*_3,1654_ = 68.77, *p* < 0.001); MCS (*F*_3,1654_ = 190.43, *p* < 0.001)).

## 4. Discussion

This study aimed to determine the severity criteria for AIS using ISI, which categorizes mild, moderate, and severe levels of insomnia. It showed that the severity criteria of the AIS were capable of categorizing insomnia severity as follows: absence of insomnia (0–5), mild insomnia (6–9), moderate insomnia (10–15), and severe insomnia (16–24).

The result of correlational analysis revealed that the AIS was strongly associated with the ISI. This finding is consistent with a previous study (*r* = 0.85) [13]. In addition, as a result of simultaneous exploratory factor analysis of all items on the AIS and ISI, it was found that nocturnal sleep disturbances and daytime sleep dysfunction were distinguished. Chronic insomnia disorder is defined as nocturnal sleep disturbances (difficulty initiating sleep, difficulty maintaining sleep, and waking up earlier than desired) and results in some form of daytime dysfunction [25]. Therefore, the findings of the two-factor structure are plausible, and it is thought that the AIS and ISI comprise almost the same construct.

The severity level of insomnia was identified using an ROC. In previous studies, some cutoff points of the AIS were shown as follows: ≥6 points (insomniac vs. non-insomniac group) [12,13], ≥7 points (insomniac vs. non-insomniac group) [9,26] and ≥10 points (insomniac vs. psychiatric patients) [12]. However, these points are a screening criterion for insomnia and do not reflect the severity of insomnia. From the results of the AUC, sensitivity, specificity, LR+, and LR−, the severity criteria of AIS could be extremely useful. Meanwhile, the sensitivity of the cutoff value between mild and moderate levels was relatively low. Since this study was based on a community sample, the validity of the cutoff value requires examination in patient samples in future research.

Furthermore, the results of ANOVAs using the severity categories of AIS or ISI were similar for changes in daytime sleepiness, depression, and health-related QoL. This is remarkable because there are no studies showing that daytime functioning declines with increasing insomnia severity. In randomized clinical trials, the severity level of insomnia (e.g., ISI ≥ 8) was used as one of the inclusion criteria of the trial [27,28] and as a remission and response criterion [29]. Nonetheless, our study is the only epidemiological study that examined the severity of insomnia, although it was shown that the prevalence of insomnia worsens daytime function such as depression and health-related QoL [19,30], and that insomnia impaired daytime function compared with good sleep [31].

This finding allows not only to examine the prevalence of insomnia in the community sample but also the prevalence, natural history, or association with daytime functions as a function of the severity of insomnia. The identification of severity of AIS in this study is important in linking the findings of epidemiological studies with those of clinical studies.

This study had some limitations. First, our participants were city government employees in Japan. Therefore, it is unclear whether they may have been representative of the general Japanese population. Second, we used cross-sectional data and, hence, we cannot provide information on the causality of the observed associations. It is necessary to plan to conduct a cohort study in this population or the general Japanese population in future research.

## 5. Conclusions

In conclusion, the results of this study showed that the AIS can be used to grade the severity of insomnia. Therefore, cross-sectional and cohort studies on the impact of insomnia on social and mental function can be studied in detail. In addition, the AIS can be used in clinical studies in future research.

## Figures and Tables

**Table 1:** Demographic characteristics and descriptive statistics of all measures.

Variables	Mean	SD
Age	45.33	12.20
Gender	Male: 39%	Female: 61%
Body mass index (BMI)	22.55	3.79
Insomnia Severity Index (ISI)	7.00	4.39
Athens Insomnia Scale (AIS)	5.02	3.60
Epworth Sleepiness Scale (ESS)	7.84	4.55
Patient Health Questionnaire-9 (PHQ-9)	4.67	4.55
Physical component summary (PCS)	47.72	7.19
Mental component summary (MCS)	47.36	7.48

**Table 2:** Exploratory factor analysis for AIS and ISI.

Items	Contents	Factor Loadings	
		Factor 1	Factor 2
Factor 1: Sleep quality and daytime function			
AIS 4	Total sleep duration	0.84	−0.15
ISI 7	Worried/distressed about sleep pattern	0.79	0.05
ISI 4	Satisfied/dissatisfied with sleep pattern	0.79	−0.05
ISI 5	Sleep problem interference with daily functioning	0.78	−0.07
AIS 5	Sleep quality	0.66	0.19
ISI 6	Sleep problem noticeable to others	0.64	−0.03
AIS 8	Sleepiness during the day	0.60	−0.08
AIS 7	Functioning capacity during the day	0.54	0.11
AIS 6	Well-being during the day	0.52	0.14
Factor 2: Nocturnal sleep			
ISI 2	Difficulty staying asleep	−0.05	0.85
AIS 2	Awakenings during the night	−0.03	0.82
ISI 3	Problem with waking too early	−0.04	0.75
AIS 3	Final awakening	−0.07	0.72
ISI 1	Difficulty falling asleep	0.11	0.51
AIS 1	Sleep induction	0.16	0.45
**Factor correlation**			
Factor 1		1.00	−0.60
Factor 2			1.00

AIS, Athens Insomnia Scale; ISI, Insomnia Severity Index.

**Table 3:** Cutoff scores of the AIS for insomnia.

Severity Level	Cutoff Score	Sensitivity	Specificity	LR+	LR−
Mild vs. moderate	6.50	0.96	0.47	1.81	0.09
	7.50	0.92	0.62	2.44	0.13
	8.50	0.84	0.75	3.30	0.22
	**9.50**	**0.74**	**0.83**	**4.45**	**0.32**
	10.50	0.64	0.89	5.89	0.41
	11.50	0.53	0.92	6.94	0.51
Moderate vs. severe	12.50	1.00	0.62	2.61	0.00
	13.50	0.88	0.76	3.61	0.17
	14.50	0.88	0.89	7.88	0.14
	**15.50**	**0.88**	**0.91**	**9.63**	**0.14**
	16.50	0.63	0.95	12.38	0.40
	17.50	0.63	0.97	20.63	0.39

AIS, Athens Insomnia Scale; LR+, positive likelihood ratio; LR−, negative likelihood ratio. Bold types indicate the cutoff values identified in this study.

## References

[1] Kim K., Uchiyama M., Okawa M., Liu X., Ogihara R. (2000). An Epidemiological Study of Insomnia among the Japanese General Population. Sleep.

[2] Okajima I., Komada Y., Nomura T., Nakashima K., Inoue Y. (2012). Insomnia as a Risk for Depression: A Longitudinal Epidemioiogic Study on a Japanese Rural Cohort. J. Clin. Psychiatry.

[3] Ohayon M.M. (2002). Epidemiology of Insomnia: What We Know and What We Still Need to Learn. Sleep Med. Rev..

[4] Riemann D., Baglioni C., Bassetti C., Bjorvatn B., Groselj D.L., Ellis J.G., Espie C.A., Garcia-Borreguero D., Gjerstad M., Gonçalves M. (2017). European Guideline for the Diagnosis and Treatment of Insomnia. J. Sleep Res..

[5] Tubbs A.S., Gallagher R., Perlis M.L., Hale L., Branas C., Barrett M., Gehrels J.-A., Alfonso-Miller P., Grandner M.A. (2020). Relationship between Insomnia and Depression in a Community Sample Depends on Habitual Sleep Duration. Sleep Biol. Rhythm..

[6] Amiri S., Behnezhad S. (2020). Sleep Disturbances and Risk of Sick Leave: Systematic Review and Meta-Analysis. Sleep Biol. Rhythm..

[7] Qaseem A., Kansagara D., Forciea M.A., Cooke M., Denberg T.D. (2016). Management of Chronic Insomnia Disorder in Adults: A Clinical Practice Guideline from the American College of Physicians. Ann. Intern. Med..

[8] Bastien C.H., Vallières A., Morin C.M. (2001). Validation of the Insomnia Severity Index as an Outcome Measure for Insomnia Research. Sleep Med..

[9] Chung K.-F., Kan K.K.-K., Yeung W.-F. (2011). Assessing Insomnia in Adolescents: Comparison of Insomnia Severity Index, Athens Insomnia Scale and Sleep Quality Index. Sleep Med..

[10] Munezawa T., Inoue Y., Morin C.M., Nedate K. (2009). Development of the Japanese Version of the Insomnia Severity Index (ISI-J). Jpn. J. Psychiatr. Treat..

[11] Soldatos C., Dikeos D., Paparrigopoulos T. (2000). Athens Insomnia Scale: Validation of an Instrument Based on ICD-10 Criteria. J. Psychosom. Res..

[12] Soldatos C.R., Dikeos D.G., Paparrigopoulos T.J. (2003). The Diagnostic Validity of the Athens Insomnia Scale. J. Psychosom. Res..

[13] Okajima I., Nakajima S., Kobayashi M., Inoue Y. (2013). Development and Validation of the Japanese Version of the Athens Insomnia Scale. Psychiatry Clin. Neurosci..

[14] Chiu H.-Y., Chang L.-Y., Hsieh Y.-J., Tsai P.-S. (2016). A Meta-Analysis of Diagnostic Accuracy of Three Screening Tools for Insomnia. J. Psychosom. Res..

[15] Buysse D.J., Reynolds C.F., Monk T.H., Berman S.R., Kupfer D.J. (1989). The Pittsburgh Sleep Quality Index: A New Instrument for Psychiatric Practice and Research. Psychiatry Res..

[16] Kaku A., Nishinoue N., Takano T., Eto R., Kato N., Ono Y., Tanaka K. (2012). Randomized Controlled Trial on the Effects of a Combined Sleep Hygiene Education and Behavioral Approach Program on Sleep Quality in Workers with Insomnia. Ind. Health.

[17] Mucsi I., Molnar M.Z., Ambrus C., Szeifert L., Kovacs A.Z., Zoller R., Barótfi S., Remport A., Novak M. (2005). Restless Legs Syndrome, Insomnia and Quality of Life in Patients on Maintenance Dialysis. Nephrol. Dial. Transplant..

[18] Hallinan R., Elsayed M., Espinoza D., Veillard A.-S., Morley K.C., Lintzeris N., Haber P. (2019). Insomnia and Excessive Daytime Sleepiness in Women and Men Receiving Methadone and Buprenorphine Maintenance Treatment. Subst. Use Misuse.

[19] Takami M., Kadotani H., Nishikawa K., Sumi Y., Nakabayashi T., Fujii Y., Matsuo M., Yamada N., the NinJaSleep Study Group (2018). Quality of Life, Depression, and Productivity of City Government Employees in Japan: A Comparison Study Using the Athens Insomnia Scale and Insomnia Severity Index. Sleep Sci. Pract..

[20] Muramatsu K., Miyaoka H., Kamijima K., Muramatsu Y., Tanaka Y., Hosaka M., Miwa Y., Fuse K., Yoshimine F., Mashima I. (2018). Performance of the Japanese Version of the Patient Health Questionnaire-9 (J-PHQ-9) for Depression in Primary Care. Gen. Hosp. Psychiatry.

[21] Takegami M., Suzukamo Y., Wakita T., Noguchi H., Chin K., Kadotani H., Inoue Y., Oka Y., Nakamura T., Green J. (2009). Development of a Japanese Version of the Epworth Sleepiness Scale (JESS) Based on Item Response Theory. Sleep Med..

[22] Fukuhara S., Suzukamo Y. (2005). Health-Related Quality of Life: The SF-8 and SF-36 Japanese Version. J. Clin. Exp. Med..

[23] Yang S., Berdine G. (2017). The Receiver Operating Characteristic (ROC) Curve. Southwest Respir. Crit. Care Chron..

[24] Edman E.W., Runge T.J. (2014). Sensitivity, specificity, LR+, and LR−: What are they and how do you compute them?.

[25] American Academy of Sleep Medicine (2014). International Classification of Sleep Disorders.

[26] Sun J.-L., Chiou J.-F., Lin C.-C. (2011). Validation of the Taiwanese Version of the Athens Insomnia Scale and Assessment of Insomnia in Taiwanese Cancer Patients. J. Pain Symptom Manag..

[27] Okajima I., Akitomi J., Kajiyama I., Ishii M., Murakami H., Yamaguchi M. (2020). Effects of a Tailored Brief Behavioral Therapy Application on Insomnia Severity and Social Disabilities Among Workers With Insomnia in Japan: A Randomized Clinical Trial. JAMA Netw. Open.

[28] Watanabe N., Furukawa T.A., Shimodera S., Morokuma I., Katsuki F., Fujita H., Sasaki M., Kawamura C., Perlis M.L. (2011). Brief Behavioral Therapy for Refractory Insomnia in Residual Depression: An Assessor-Blind, Randomized Controlled Trial. J. Clin. Psychiatry.

[29] Epstein D.R., Sidani S., Bootzin R.R., Belyea M.J. (2012). Dismantling Multicomponent Behavioral Treatment for Insomnia in Older Adults: A Randomized Controlled Trial. Sleep.

[30] Komada Y., Nomura T., Kusumi M., Nakashima K., Okajima I., Sasai T., Inoue Y. (2012). A Two-Year Follow-up Study on the Symptoms of Sleep Disturbances/Insomnia and Their Effects on Daytime Functioning. Sleep Med..

[31] LeBlanc M., Mérette C., Savard J., Ivers H., Baillargeon L., Morin C.M. (2009). Incidence and Risk Factors of Insomnia in a Population-Based Sample. Sleep.

